# The Argonaut

**Published:** 1890-12

**Authors:** 


THE ARGONAUT. Published quarterly by Kent B. Waite, A. M., M. D., 106 Euclid Avenue, Cleveland, Ohio. Subscription price, $1.00 per year.

This is a new medical journal published in the interest of the new medical college at Cleveland, Ohio, which has been started in opposition to the Cleveland Homœopathic Hospital College. The new journal contains about twenty pages and is full of local news of the college of which it is the adherent.

It is greatly to be regretted that the physicians of Cleveland should deem it necessary to start another college. As the old college had resolved to teach pure Homœopathy it certainly is doing all that can be expected of it. It should then have been cordially sustained by the homœopathic physicians of Cleveland and not hampered by the rearing of a second college that must only divide with it the sustenance that was hardly sufficient for the one.

